# Identification of a novel nucleolin related protein (NRP) gene expressed during rat spermatogenesis

**DOI:** 10.1186/1471-2199-10-64

**Published:** 2009-07-01

**Authors:** Keerthi T Chathoth, Gayatri Ganesan, MRS Rao

**Affiliations:** 1 Molecular Biology and Genetics Unit, Jawaharlal Nehru Centre for Advanced Scientific Research, Jakkur, Banglore, India, 560064; 2 Department of Biochemistry, Indian Institute of Science, Bangalore, India, 560012

## Abstract

**Background:**

Nucleolin is a major nucleolar phosphoprotein involved in various steps of ribosome biogenesis in eukaryotic cells. As nucleolin plays a significant role in ribosomal RNA transcription we were interested in examining in detail the expression of nucleolin across different stages of spermatogenesis and correlate with the transcription status of ribosomal DNA in germ cells.

**Results:**

By RT PCR and western blot analysis we found that nucleolin is strongly down regulated in meiotic spermatocytes and haploid germ cells. We have identified a new nucleolin related protein (NRP) gene in the rat genome, which is over expressed in the testis and is up regulated several fold in meiotic spermatocytes and haploid germ cells. The NRP protein lacks the acidic stretches in its N terminal domain, and it is encoded in rat chromosome 15 having a different genomic organization as compared to nucleolin gene present on chromosome 9. We have also found NRP genes encoded in genomes of other mammalian species. We performed run-on transcription assay where we have observed that rDNA is transcribed at much lower level in meiotic spermatocytes and haploid spermatids as compared to diploid cells. By siRNA knock down experiments we could also demonstrate that NRP can support rDNA transcription in the absence of nucleolin.

**Conclusion:**

We have identified a new nucleolin variant over expressed in germ cells in rat and analyzed its domain structure. We attribute that the transcriptional activity of rDNA genes in the late spermatogenesis is due to the presence of this variant NRP. The expression of this variant in the germ cells in the absence of nucleolin, could have additional functions in the mammalian spermatogenesis which needs to be investigated further.

## Background

In eukaryotic cells, the nucleolus is the site of ribosome biogenesis, which includes transcription of ribosomal DNA, processing of precursor rRNA and pre-ribosome assembly [1-3]. The rate of synthesis of ribosomal RNA varies depending upon the proliferative status of the cell and hence is accentuated in cancer cells [4]. The ribosomal DNA (45S precursor including 18S, ETS, ITS and 28S and 5.8S) is transcribed by RNA polymerase I and 5S rRNA is transcribed by RNA polymerase III in the nucleolus. The ribosomal protein genes are transcribed by RNA polymerase II in the nucleoplasm and after synthesis in the cytoplasm are transported into the nucleolus for pre-ribosome assembly. Several proteins and small nucleolar RNAs are involved in various steps of ribosome biogenesis. Among these, nucleophosmin (B23) and Nucleolin (C23) are the two most abundant non- ribosomal proteins whose critical functions are still being elucidated [5-7]. Both these proteins, especially nucleolin, also undergo several modifications like phosphorylation [8,9] methylation [10] and ADP-ribosylation [11] for regulating their functions.

The mammalian nucleolin is of 75–77 kDa showing an apparent molecular mass of 100–110 kDa because of its aberrant mobility in an SDS-polyacrylamide gel. The nucleolin protein is made up of three structural domains. The first N-terminal one third of the protein contains a contiguous stretch of highly acidic amino acids interspersed with basic amino acids. This domain also contains several phosphorylation sites for casein kinase II [12,13], p34cdc2 [14] and protein kinase C [15]. The central domain consists of four RNA binding domains called RRM. It is generally believed that these four RRMs have arisen by a duplication of the 2 RRM domains. The C-terminal domain is rich in glycine, arginine and phenylalanine residues, which is known as the GAR domain. The function of this GAR domain is still not clear and it is believed that this domain facilitates the interaction of nucleolin with several other RNA binding proteins including ribosomal proteins in addition to rRNA itself [16]. Nucleolin is predominantly localized to fibrillar component around the fibrillar centers with a small proportion also being present in the granular compartment of the nucleolus [17]. Recently nucleolin has also been detected on cell membranes [18]. Nucleolin can be classified under multifunctional proteins having a variety of functions at different steps of ribosome biogenesis. For example it has been shown to have both stimulatory and repressive role in rDNA transcription [19,20]. The N-terminal acidic domain has been shown to be involved in pre-rRNA processing [21] and histone chaperone activity [22]. Nucleolin is conserved in several species including plants [23,24], *Xenopus *[25,26] and yeast [27] with a little variation in the N terminal domain, RRM motifs and in the length of the RGG stretch at the C-terminus and they have been termed as Nucleolin like proteins [28].

Mammalian spermatogenesis is a fascinating model of cellular differentiation process encompassing several rounds of mitotic division of spermatogonia, meiotic division and maturation of haploid spermatids during the spermiogenesis process. The rate of ribosomal RNA synthesis dramatically changes during this long process of one round of germ cell differentiation. Very early autoradiographic studies have shown that spermatogonia are very active in rRNA synthesis, which peaks at the mid-pachytene level [29]. Using several cytological and immunochemical techniques it has been shown that there is also extensive morphological change in the nucleolar structure during different stages of spermatogenesis [30]. Recently, a spermatogenesis specific variant of *Drosophila *nucleolin, *Modulo*, has been described whose expression preceeds the spermatid differentiation process [31]. Modulo also acts as a transcription factor regulating both the meiotic arrest genes and the spermatid differentiation genes. While analyzing the detailed expression pattern of nucleolin during different stages of rat spermatogenesis, we discovered a new germ cell specific nucleolin gene, which is encoded in rat chromosome 15. The detailed characteristics of this Nucleolin related protein (NRP) and its role in rDNA transcription is reported in this communication.

## Results

### Nucleolin is down regulated in meiotic spermatocytes and haploids

In one of our preliminary studies on the genome wide gene expression pattern among gametic diploid, meiotic spermatocytes and spermatid cells using UHN microarray slides we observed that nucleolin is highly down regulated in meiotic spermatocytes and haploid spermatogenic cells (Sneha and M.R.S.Rao, unpublished data). Nucleolin is a major non-ribosomal phosphoprotein present in the nucleolus of a eukaryotic cell [32]. Its expression pattern is well correlated with the Pol I mediated transcription of ribosomal RNA gene [20]. As nucleolin plays a significant role in ribosomal RNA transcription we were interested in examining in detail the expression of nucleolin across different stages of spermatogenesis and correlate with the transcription status of ribosomal DNA in germ cells.

**Figure 1:**
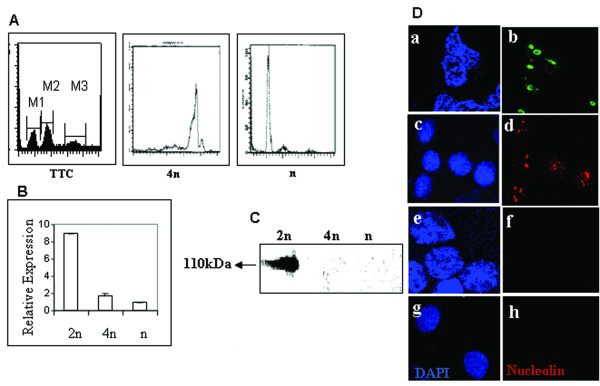
**Expression pattern of nucleolin in spermatogenic cells**. A) FACS profile of total testicular cells (TTC), meiotic spermatocytes; (4n); and spermatid cells (n) purified from rat testis. **B) **Real time PCR analysis of nucleolin mRNA normalized with respect to Naca1. Differences (*n*-fold) in expression of nucleolin were compared across gametic diploid cells, meiotic spermatocytes and spermatid cells keeping low expressing cells as the reference. Each value plotted represents an average of 3 independent experiments ± standard deviation. 2n, gametic diploid cells (testicular cells from 10 day old rat); 4n, meiotic spermatocytes; and n, spermatids. **C) **Western blot analysis of nucleolin using mouse monoclonal nucleolin antibody using goat anti-mouse IgG HRP conjugate as secondary antibody. 100 μg of protein was loaded in each lane. 2n, diploid cells; 4n, meiotic spermatocytes; and n spermatid cells. **D) **Immunolocalisation of nucleolin using mouse monoclonal nucleolin antibody, followed by counterstaining with goat anti-mouse Alexa 568 and 488. Nucleus was stained with DAPI (1 μg/ml). HeLa cells, (panel a-b); gametic diploid cells, (panel c-d); meiotic spermatocytes, (panel e-f); and spermatid cells, (panel g-h).

To begin with we carried out real time PCR analysis for nucleolin messenger RNA in spermatogonial cells (2n), meiotic spermatocytes (4n), and haploid spermatids (n). The meiotic spermatocytes and spermatid cells were isolated using centrifugal elutriation technique and the relative purity of these cells as monitored by FACS analysis were found to be 75% and 95% respectively as shown in Figure 1A. It was evident that nucleolin was drastically down regulated at RNA level both in meiotic spermatocytes as well as haploid germ cells (Figure 1B). The nucleolin transcript was however detected in actively dividing gametic diploid spermatogonial cells. The absence of nucleolin expression in meiotic spermatocytes and spermatid cells was further corroborated at the protein level by carrying out western blotting analysis of total nuclear proteins probed with monoclonal anti-nucleolin antibodies (Santa Cruz). As can be seen from Figure 1C, a band around 110 kDa was detected in diploid germ cells but not in meiotic spermatocytes and haploid spermatid cells. We further confirmed the absence of nucleolin by indirect immunoflourescence method using the monoclonal anti-nucleolin antibody. We used HeLa cells as one of the control where we observed nucleolin to be localized in the dark nucleolar region (Figure 1D a–b) as expected. We could also localize nucleolin in nucleolar structures in nuclei of spermatogenic diploid cells (panel c-d). However, we could not see any nucleolin staining in both the meiotic spermatocytes (panels e-f) and haploid germ cells (panel g-h). Thus it was quite clear from the above observation that nucleolin, one of the major non-ribosomal proteins involved in ribosome biogenesis, was strongly down regulated at both RNA and protein level in meiotic as well as post meiotic germ cells.

### Fibrillarin and UBF are expressed throughout spermatogenesis

**Figure 2:**
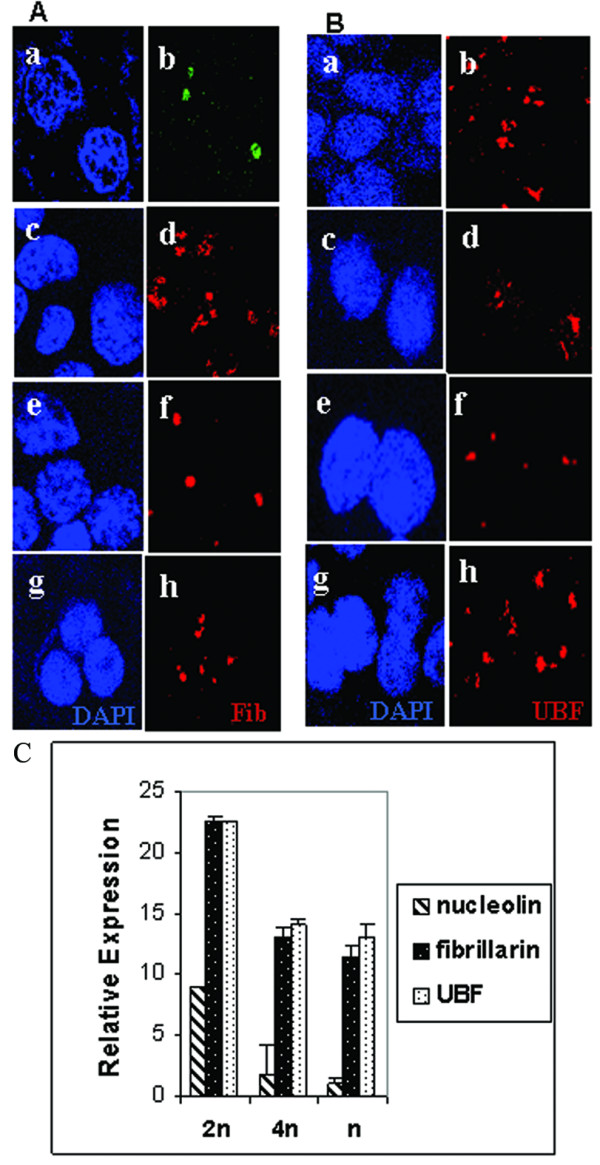
**Expression of fibrillarin and UBF in spermatogenic cells**. **A) **Immunolocalisation of fibrillarin using goat polyclonal anti-fibrillarin. Nucleus was stained with DAPI. HeLa cells, (panel a-b); gametic diploid cells, (panel c-d); meiotic spermatocytes, (panel e-f); and spermatid cells, (panel g-h). B) Immunolocalisation of UBF using rabbit polyclonal UBF antibody followed by goat anti-rabbit conjugated with Alexa 568 as secondary antibodies. Nucleus was stained with DAPI. HeLa cells, (panel a-b); gametic diploid cells, (panel c-d); meiotic spermatocytes, (panel e-f); spermatid cells, (panel g-h). C) Realtime PCR of nucleolin, Fibrillarin and UBF. Differences (*n*-fold) in expression of nucleolin, fibrillarin and UBF were compared in meiotic spermatocytes, spermatids and gametic diploid cells keeping the low expressing cells as the reference. Each value plotted represents an average of 3 independent triplicate experiments ± standard deviation. 2n, diploid cell (striped bar); 4n, meiotic spermatocytes (black bar with white dots); and n, haploids cells (white bar with black dots).

In addition to nucleolin two other proteins that are intimately involved in rDNA transcription and processing are UBF and Fibrillarin. Fibrillarin is a 36 kDa protein that is involved in rRNA maturation and assembly [33]. UBF is an upstream binding factor of 108 kDa and is a major transcription factor essential for the polymerase I dependent transcription [34]. We were curious to examine the expression of these two proteins in the meiotic spermatocytes and spermatids cells. For this purpose we carried out immunoflourescence assay using anti-fibrillarin and anti-UBF antibodies (Santa Cruz) and the results are shown in Figure 2. It was interesting to see that both Fibrillarin (Figure 2A) and UBF (Figure 2B) were detectable in meiotic spermatocytes (panels e-f) as well as haploid germ cells (panels g-h). Panels (a-b) and (c-d) show the localization pattern of these proteins in HeLa and gametic diploid cells respectively. In order to get a more quantitative picture of the expression of these two proteins, we carried out real time PCR analysis of Fibrillarin, UBF and also that of nucleolin in these germ cells. The expression level of Fibrillarin and UBF were reduced by approximately less than one fold in meiotic spermatocytes and by two fold in haploid cells as compared to gametic diploid cells while nucleolin was strongly down regulated (Figure 2C). Above results showed that unlike nucleolin other nucleolar proteins essential for RNA polymerase I transcription and processing are present in the testicular germ cells irrespective of the stages of differentiation. However, a search of the rat genome data base for fibrillarin identified a variant of fibrillarin gene on rat chromosome 4. The expression of this gene in testis or other somatic tissues is not known and has to be investigated.

### Transcription of rDNA in haploid spermatids

**Figure 3:**
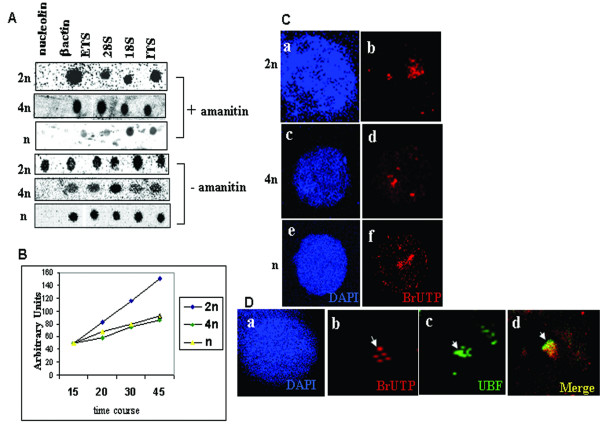
**Transcription of rDNA in spermatogenic cells**. A) Nuclear run-on transcription in nuclei of different spermatogenic cells in the presence (top 3 panels) and absence of α-amanitin (bottom 3 panels). Labelled transcripts were hybridised with each of the domains of cold pre-rRNA and nucleolin. 2n, diploid cells; 4n, meiotic spermatocytes; and n, spermatid cells. Lane 1-nucleolin; lane 2-beta actin; lane 3-External transcribed spacer (ETS); lane 4–28S rRNA; lane 5–18S rRNA; lane 6-Internal transcribed spacer (ITS). B) Rate of rDNA transcription assayed by nuclear run-on transcription in diploid, meiotic spermatocytes and spermatid cells. RNA isolated at different time periods were hybridised to ETS. Each value represents an average of two independent experiments. Line with blue icon, gametic diploid cells; green, meiotic spermatocytes and yellow, spermatid cells. C) *In situ *run on transcription assayed in the presence of α-amanitin. BrUTP labelled transcripts were visualized using mouse monoclonal anti-BrdU. Nucleus is stained with DAPI. 2n, gametic diploid cells (panel a-b); 4n, meiotic spermatocytes (panel c-d); and n spermatid cells (panel e-f). D) Colocalisation of BrUTP labelled transcript with UBF in round spermatids in the presence of α-amanitin. Transcripts were detected by monoclonal anti-BrdU and UBF with anti-UBF a) DAPI stained nuclei; b) BrUTP labelling; c) UBF localisation; and d) merge of b and c.

As mentioned in the introduction, Nucleolin plays a major role in plethora of rRNA processing activities including rDNA transcription, [35-38], we were interested to study the rDNA transcription status in different germ cells. For this purpose we initially carried out *in vitro *nuclear run-on transcription in meiotic spermatocytes and haploid cells using gametic diploid germ cells from 10-day-old rat as control. A run-on transcription assay was carried out by labeling the nascent rRNA transcripts with α^32^P UTP both in the presence as well as in the absence of α-amanitin (100 μg/ml) to score specifically for Pol I mediated transcription. The results of such an experiment are shown in Figure 3A. The presence of run on transcripts for ribosomal RNA (28S, 18S, ETS, and ITS) were detected in both α-amanitin treated as well as untreated cells of 2n, 4n and n whereas transcripts of Pol II products such as β actin and Nucleolin were detected only in the absence of α-amanitin. In conformity with our observation made above, nucleolin nascent transcript was observed only in diploid cells but was absent in meiotic spermatocytes and spermatid cells even in this run-on transcription assay. We also examined the rate of transcription of ribosomal RNA as calculated from the amount of radioactivity incorporated as a function of time which is graphically represented in Figure 3B. It is clear from the figure that rDNA genes are indeed transcribed, showing a linear increase as a function of time in all the three type of germ cells. However, it can be noticed that both the rate and the extent of rDNA transcription in meiotic spermatocytes and spermatid cells are much lower than in gametic diploid cells.

In order to extend this observation further, an *in situ *run-on transcription was carried out in meiotic spermatocytes and spermatid cells by the incorporation of BrUTP in the presence of α-amanitin followed by staining with monoclonal anti BrdU antibodies. Interestingly, fluorescent signal representing nascent rRNA transcripts were detected in both meiotic spermatocytes (panel c-d) as well as in spermatid cells (panel e-f) confirming the results of *in vitro *nuclear run-on transcription. As mentioned earlier UBF is a polymerase I specific transcription factor and hence we examined the colocalisation pattern of UBF with these rDNA transcripts. As can be seen in Figure 3D, we did observe the colocalisation of UBF protein with BrUTP signal, proving the authenticity of rDNA transcription that we were detecting in the meiotic spermatocytes and spermatid cells. Thus both the *in vitro *and *in vivo *run-on experiments proved unambiguously that rDNA transcription does take place in the meiotic spermatocytes and spermatid cells where nucleolin was not detected.

### A nucleolin related protein (NRP) is expressed in meiotic and post meiotic germ cells

**Figure 4:**
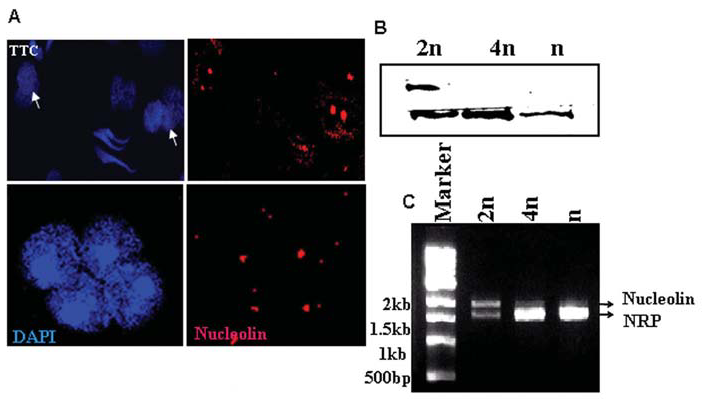
**Presence of nucleolin like variant (NRP) in spermatogenic cells**. A) Immunolocalisation of nucleolin using goat polyclonal anti-nucleolin followed by alexa dye conjugated anti goat in round spermatids. Haploid cells, (panel a-b); and meiotic spermatocytes, (panel c-d). White arrows represent the round spermatid cells. B) Western blot analysis of nucleolin using rabbit polyclonal anti-nucleolin antibodies. 2n, diploid germ cells; 4n, meiotic spermatocytes and n spermatid cells. 2n diploid cells show two bands in contrast to the one band shown in 4n meiotic spermatocytes and n spermatid cells. C) Semiquantitative RT-PCR of full-length nucleolin mRNA. Lane 1 – marker; lane 2 – 2n, diploid germ cell; lane 3 – 4n, meiotic spermatocytes; and lane 4 – n, spermatid cells.

**Figure 5:**
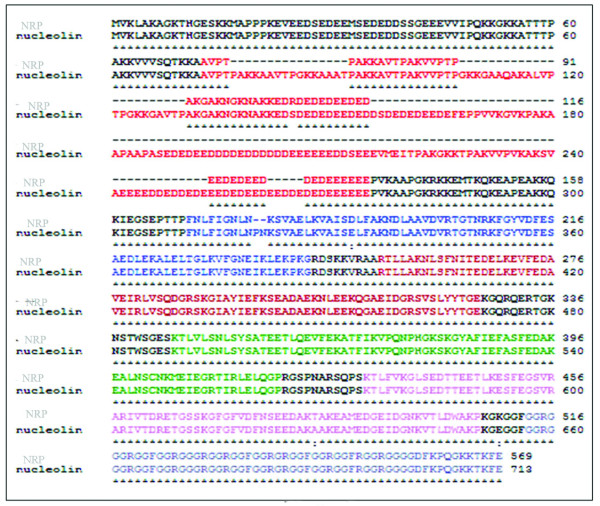
**Amino acid sequence comparison of nucleolin and NRP**. Amino acid alignment of rat nucleolin and NRP using software ClustalW. Red coloured region is the N-terminal acidic stretch. Blue colour, RRM1; dark red, RRM2; green, RRM3; pink, RRM4 and violet, C terminal GAR domain. The dotted lines show the missing stretches of amino acid in the N terminal of NRP comprising of acidic domain.

In the backdrop of the essentiality of nucleolin in rDNA transcription [20], we were surprised to observe rDNA transcription in both the meiotic spermatocytes and spermatid cells although at much reduced level. In all of our experiments, described above, we had used monoclonal antibodies against nucleolin (Santa Cruz). For curiosity we then used polyclonal antibodies against nucleolin (a gift from Dr. Philip Bouvet) and repeated the immunoflourescence analysis. To our surprise we did observe foci lighted by polyclonal nucleolin antibody in both meiotic spermatocytes and spermatid cells (Figure 4A). Subsequently we went ahead with western blot analysis of the nuclear proteins with these polyclonal anti-nucleolin antibodies. As can be seen in Figure 4B these antibodies lighted up two polypeptide bands in gametic diploid cells (2n) while only the bottom band was observed in meiotic spermatocytes (4n) and spermatid (n) cells. The upper band in the gametic diploid cells corresponds to 110 kDa region and represents nucleolin, which is absent in meiotic spermatocytes and spermatid cells. The presence of a lower band suggested to us the possible existence of a nucleolin variant in the germ cells. To further verify this possibility we amplified nucleolin mRNA from diploid germ cells by using the ORF specific forward and reverse primers (for primer sequences see additional file 1). The analysis of PCR products shown in Figure 4C clearly show that two amplicons were observed in gametic diploid cells while only one amplicon of lower size of around 1.6 kb was observed in meiotic spermatocytes and spermatid cells. Each of these two bands was eluted and the DNA sequence was determined. The sequence of the upper 2 kb band exactly corresponded to nucleolin sequence and the lower band of 1.6 kb corresponded to a new variant with a sequence very similar to nucleolin which we refer here as nucleolin related protein (NRP). The amino acid sequence alignment of rat nucleolin and the NRP is shown in Figure 5. The sequences are almost identical (88%) except that the amino acid stretches (1) ^76^PAKKAAVTPGKKAAAT^92^, (2)^107^GKKGAAQAKALVPTPGKKGAVTP^130^, (3) ^156^DSDEDEDEEDEFEPPVVKGVKPAKAAPAAPASEDED EEDDDDEDDDDDDEEEEEEDDSEEEVMEITPAKGKKTPAKVVPVKAKSVAEEEEDDEDDEDE^253^,(4) ^261^EEDDE^266^, (5) ^319^PN^321 ^from that of nucleolin were missing in NRP. A BLAST search of the NRP sequence in the rat genome database Ensemble identified the NRP as a predicted gene on chromosome 15. This nucleolin related protein gene in chromosome 15 has been reported as a Ncl-Ps1 referring to nucleolin pseudogene in the rat genome database (Entrez GeneID:64556). Since we observe the expression at protein level this gene is now interpreted as expressed gene and has now been named as nucleolin related protein (NRP) with Entrez accession number: FJ817497.

**Figure 6:**
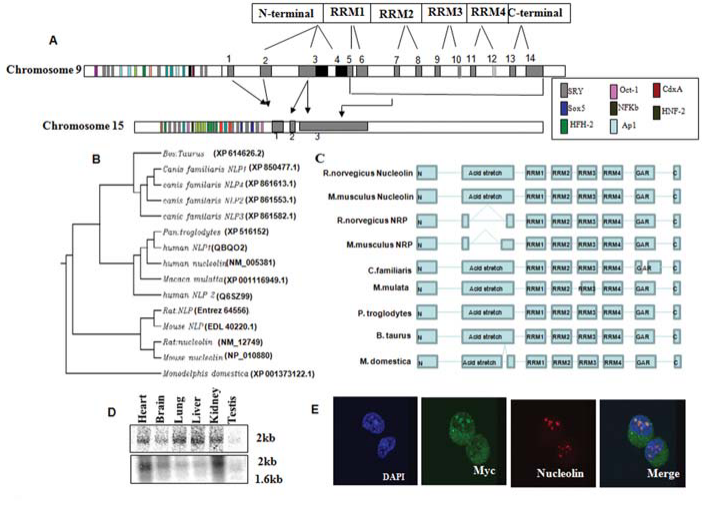
**Genomic organization and evolutionary relationship of nucleolin and NRPs in different eutherian mammals**. A) Schematic representation of genomic organization of *nucleolin *on (chromosome 9) and *NRP *gene on (chromosome 15). Exons are represented in grey bars interrupted with white bars as introns. The black region of 3^rd ^exon and complete exon 4 of nucleolin are absent in the *NRP *gene and the arrow represents the corresponding exons of *nucleolin *found in *NRP*. The colored bars in the upstream sequences represent the different transcription factor binding sites. B) Conservation of the NRP across 8 different mammals. ClustalW was used to generate the dendrogram. C) Domain alignment of Nucleolin and NRP from different species showing the missing stretch of acidic amino acids in mouse and rat NRP as against nucleolin of other species. D) Multiple tissue northern blot analysis using rat polyA RNA blot. Upper panel, hybridisation with a probe specific to nucleolin spanning the exon 4. Bottom panel, hybridisation with full length nucleolin common to both. E) Colocalisation of nucleolin and NRP. pCMV myc-NRP was transfected into GC1-spg cells. Anti Myc was used to detect NRP in the transfected cells, while the endogenous nucleolin was detected by polyclonal nucleolin antibody followed by appropriate secondary. Panel (a), nucleolin; panel (b), myc; and panel (c), merge of a and b.

For further validation, NRP gene of 3 kb and nucleolin gene (1 kb spanning 3^rd ^and 4^th ^exon with an intron in between) was amplified, gel eluted and sequenced (using primer pairs (13) and (14) respectively as given in the additional file 1). Both the genes were amplified from the same genomic DNA template, thus experimentally proving the authenticity of their gene sequence present in the database. A detailed analysis of the gene structure of this new NRP gene resident on chromosome 15 revealed that it consists of three exons and two introns as shown in Figure 6A as against the presence of 14 exons and 13 introns of the nucleolin gene resident on chromosome 9. The important features of the NRP gene in comparison with the nucleolin gene are a) A small region of exon 1 and complete exon 2 of nucleolin forms the exon 1 of NRP. b) A region of exon 3 of nucleolin, 37 bp long, forms the exon 2 of NRP. c) A portion of exon 3 of nucleolin and complete exon 5 through 14 of nucleolin are fused in NRP as one large 3rd exon. But the sequence spanning the 3'UTR of both the genes are identical. All together NRP has acquired all the exons of nucleolin except the missing sequences mentioned but no introns. The splice signal (GU/AG) is found to be present in the exon/intron boundary of the NRP variant as predicted in the database except for one intron. It is predicted that the NRP has 3 introns and the 3^rd ^intron, is supposed to be a single nucleotide C, but we have found that C is a part of the exon and it is present in the mRNA transcript of the variant. We have seen all the sequence characteristics of nucleolin in the variant NRP except the acidic stretch, we are still unsure of the exact mRNA 5' and 3' nucleotide. However, the northern blot shows only a band of 1.6 kb in length thus eliminating the possibility of the presence of additional exons. The domain structure of nucleolin encompasses an N-terminal domain (acidic stretch), four RRM (RRM1, RRM2, RRM3 and RRM4) domains and C-terminal GAR domain rich in glycine and arginine as depicted in (Figure 6A). The exon 3 of NRP together constitutes both the GAR domain and the RNA binding domains which are actually encoded by exons 5 through 14 in nucleolin. It is interesting to note that the monoclonal antibodies that we procured from Santa cruz was raised against the long acidic stretch region which is absent in the NRP and hence explains the inability of detection of the NRP in our experiments using these monoclonal antibodies. In addition to the coding sequence, we also looked into the 5' proximal promoter region of rat nucleolin and NRP gene for possible conserved and also divergent sequence elements by using the TF search and promoter inspector programmes. Apart from the common transcription factors that bind to the promoter region of both NRP and nucleolin like HFH, SRY, CdxA, NFkB, AP1, Oct-1, HNF-2 etc, a unique binding site for Sox 5 adjacent to tandem repeats of SRY binding site is present in the NRP promoter region, which is absent in the promoter region of nucleolin. Sox 5 is reported to be a testis specific transcription factor that acts cooperatively with SRY [39] and therefore possibly explains the up regulation of NRP in meiotic and post meiotic germ cells. We would also like to point here that NRP gene is also expressed in gametic diploid cells but is highly up regulated in meiotic spermatocytes and spermatid cells (Figure 4C).

### Evolutionary relationship among mammalian nucleolin and NRP

We then analyzed the published sequence database of other mammalian species to see whether NRPs are also encoded in their genome and also constructed a phylogenetic tree to understand the evolutionary relationship among these nucleolin family members (Figure 6B). We detected one novel NRP in the mouse genome, which is identical to the rat NRP in addition to the regular nucleolin gene on mouse chromosome 1. This NRP is reported as pseudogene in the mouse genome database on chromosome 11 and chromosome 1. Sequencing of the nucleolin and NRP amplified from RNA of mouse GC1-Spg cells showed that the mouse NRP is almost 90% identical to rat NRP sequence. However the nucleotide sequence did not exactly correspond to the either of the pseudogenes of mouse but was found to be identical to a predicted splice variant of nucleolin mRNA itself present on chromosome 1. There is a complete absence of 3^rd ^exon of nucleolin mRNA in the case of mouse leading to the lack of the acidic stretches, showing that the predicted nucleolin spice variant in mouse is actually expressed in mouse spermatogonial germ cells (data not shown). When we compared the amino acid sequences of human NRP1 (QBQO2) and NRP2 (Q6SZ99) on chromosome 2 with that of human *nucleolin *on the same chromosome, we observed that NRP1 lacks a portion of N-terminal domain, complete RRM1 domain and a portion of RRM2 while NRP2 lacks a portion of N-terminal domain but retains the RRM domains. The *Canis familaris *genome also encodes 4 NRP genes. The human *nucleolin*/*NRP *genes have separately evolved compared to the rodent *nucleolin*/*NRP *gene while the *Canis familaris *NRPs segregate separately. From above observations it is obvious that NRPs do exist in different mammalian species. The domain wise alignment of the nucleolin and NRP from different species indicates that except for the acidic stretch that is absent in the NRPs other major domains such as RRM1–4 and the GAR domains are very well conserved (Figure 6C)

### NRP is over expressed in germ cells and localizes to nucleolus

We were then interested to study the expression pattern of the rat NRP gene across many other tissues. A northern blot analysis of nucleolin and NRP was carried out in major tissues of the rat using a Multiple Tissue Northern blot obtained from Clonetech. *nucleolin *was specifically probed using the probe that spans the 4^th ^exon using primer pairs (5) (see additional file 1), which is missing in NRP. As seen from Figure 6D top panel, the *nucleolin *specific signal of 2 kb was present in all the tissues including testis. A faint signal in the testis (total testicular RNA) is due to the presence of RNA from diploid cells. Since the sequence of *NRP *is very much similar to nucleolin it was difficult to design specific primers for *NRP*. Therefore, a common probe (full length *nucleolin *mRNA) was used that can detect both *nucleolin *and *NRP *using primer pairs (12) (see additional file 1) which showed the presence of two bands in testis and 2 kb corresponding to the size of nucleolin in other tissues (Figure 6D bottom panel). However, a very faint signal in other tissues was also detected. The functional significance of low level of expression of NRP in somatic tissues and its up regulation in meiotic and post meiotic germ cells remains to be explored. It is quite likely that the presence of Sox 5 binding element in the promoter sequence determines the higher level of NRP expression in testis in the absence of nucleolin. We were curious to know whether NRP also gets localized specifically to nucleolus. For this purpose we cloned the rat NRP cDNA sequence in a pCMV vector with a c-Myc epitope at its N-terminal end. After transfection in GC1-Spg cell line (derived from B type spermatogonial cells), the localization was detected by indirect immunoflourescence assay using the monoclonal anti-myc antibodies. As can be seen in Figure 6E the NRP also specifically colocalises to nucleolus along with endogenous nucleolin probed with polyclonal anti-nucleolin antibodies. Thus all these observations suggest that NRP is over expressed in testis in the absence of nucleolin and it is found to be localized in the nucleolus.

### NRP supports rDNA transcription in the absence of nucleolin

**Figure 7:**
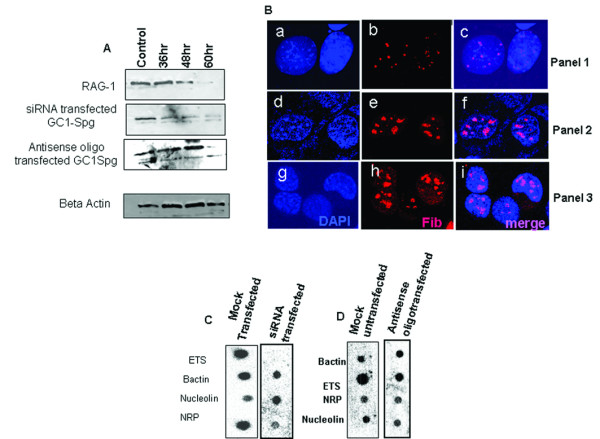
**NRP supports rDNA transcription**. A) Western blot analysis of nucleolin in GC1-Spg cells after siRNA and antisense oligo transfection using rabbit polyclonal nucleolin antibody. Panel 1, RAG cell line (siRNA transfected) and panel 2, GC1-Spg (siRNA transfected) and panel 3 GC1-Spg (antisense oligo transfected) – lane 1, (24 hr); lane 2, (36 hr); lane 3, (48 hr); and lane 4 (60 hr). B) Immunolocalisation of fibrillarin in GC1-Spg cell line after siRNA and antisense oligo transfection. GC1-Spg cells after 60 hour post transfection with siRNA and antisense oligo were fixed and stained with anti-fibrillarin antibody and further with anti-goat secondary antibody conjugated with alexa red. Panel1, (siRNA transfected) and panel 2, (antisense oligo transfected) and panel 3, (control). C) Nuclear run on transcription in GC1-Spg cell line after siRNA transfection. Cells after 60 hours post transfection with siRNA were harvested and nuclear run-on transcription was carried out as described earlier. Lane 1, untransfected and lane 2, transfected. D) Nuclear run on transcription in GC1-Spg cell line after antisense oligo transfection. Cells after 60 hours post transfection with antisense oligo were harvested and nuclear run-on transcription was carried out. Lane 1, untransfected and lane 2, transfected.

The experiment described above showed that rDNA is transcribed in meiotic spermatocytes and spermatid cells in the absence of nucleolin. One can argue that some other proteins other than NRP may be responsible for the rDNA transcription that we are detecting in meiotic spermatocytes and spermatid cells. In order to demonstrate that NRP can support the rDNA transcription, we carried out knock down experiment in GC1-Spg cell line (derived from B type spermatogonial cell). We had to use these cell lines but not the meiotic spermatocytes and spermatid cells because the transfection technique for mammalian germ cells is not available at present. We performed siRNA mediated down regulation of nucleolin and NRP (smart pool, Dharmacon) in mouse GC1-Spg and RAG-1 (Renal adenocarcinoma) cell lines. As seen from the western blot image (Figure 7A), a down regulation of nucleolin is seen in a time dependent manner in RAG-1 cells which is a somatic cell line that does not express NRP. In GC1-Spg cells siRNA mediated down regulation resulted in down regulation of both nucleolin as well as NRP as probed by a western blot analysis (The siRNA pools contain target sequences spanning the entire length of nucleolin which is also present in NRP). Further, we specifically knocked down the expression of the nucleolin by using antisense LNA oligos (oligo a and oligo b from Exiqon given in additional file 1) that were designed against the fourth exon region which is unique to nucleolin and absent in NRP. Interestingly this down regulation resulted in decreased intensity of nucleolin band in the western analysis whereas the intensity of the NRP band remained unaffected. This also confirms to our early observations that the two proteins differ only in their 4^th ^exon region. β actin was used a loading control whose protein level remained unchanged.

We then looked into the nucleolar morphology in siRNA and antisense RNA transfected GC1-Spg cells. Immunoflouresence was performed using fibrillarin antibody after 60 hours of post transfection followed by Alexa conjugated secondary antibody (Figure 7). As can be seen from the Figure 7B multiple micro nucleoli were observed in siRNA silenced cells where both nucleolin and NRP levels are down regulated. These observations are similar to those reported by Ugrinova *et al *[40]. On the other hand in antisense oligonucleotide treated cells (panel 2) the nucleolar architecture remained more or less similar to the control sets (panel 3).

We next tried to address the status of rDNA transcription under these silenced conditions. Nuclear run-on transcription assays were carried out in both siRNA treated cells (Figure 7C) and antisense oligonucleotide treated GC1-Spg cells (Figure 7D). In siRNA treated cells where both nucleolin and NRP levels are down regulated, the rDNA transcription was also greatly reduced when compared to lipofectamine transfected control cells. β actin remained constant both in control as well as siRNA transfected run-on experiments. Similar experiment carried out in antisense oligonucleotide treated cells (Figure 7D) showed that the rDNA transcription was similar to lipofectamine transfected control cells. The experiment above thus suggests a possible role of the variant nucleolin in rDNA transcription even in the absence of nucleolin.

## Discussion

The spermiogenesis process in mammals involves extensive reorganization of nuclear morphology of haploid spermatids generating highly condensed and transcriptionally inactive testicular spermatozoa. Transcriptional activity of haploid spermatids is now fairly documented. Some of the early observation based on ultra structural studies and in situ labeling techniques have shown that although ribosomal RNA can be detected in round spermatids [41] there is a continuous nucleolar inactivation during the spermiogenesis process [42]. Many protein coding genes transcribed by RNA Pol II are stored as ribonucleoprotein particles which are subsequently activated for translation during late stages of spermiogenesis [43]. Based on the circumstantial evidence it is generally believed that preformed ribosomes which are passed on from one meiotic prophase spermatocytes to haploid round spermatids support protein synthesis of the newly transcribed protein coding genes. However, there has been no systematic molecular study on the status of ribosomal RNA synthesis and processing in the haploid germ cells. The present study was initiated based on our preliminary observation that nucleolin is strongly down regulated in both meiotic spermatocytes and haploid round spermatids. Nucleolin is a major nucleolar phosphoprotein which is involved in various steps of ribosome biogenesis in the nucleolus (7).

A detailed study on the expression pattern of nucleolin gene by real time PCR, immunofluoroscence and western blot analysis has clearly demonstrated that nucleolin expression is strongly reduced both in meiotic spermatocytes and spermatid cells. Interestingly we have discovered the presence of a nucleolin related protein gene encoded on rat chromosome 15 which is expressed only in testicular germ cells and is highly up regulated in meiotic spermatocytes and spermatid cells. The germ cell specific up regulation of the NRP gene is probably determined by the presence of Sox 5 binding element only in the 5' upstream sequence of the NRP gene. Sox 5 is an important transcription factor determining the testis expression of a particular gene [39].

Our experiment described here clearly demonstrated that rDNA is transcribed both in meiotic spermatocytes and spermatid cells although at 50% less compared to gametic diploid spermatogonial cells. Based on our present understanding of the functional role of the different domains of nucleolin we would like to interpret the findings reported in the present communication as follows. The nucleolin related protein (NRP) which is present in the meiotic spermatocytes and spermatid cells supports rDNA transcription in these cells. We confirmed this role of NRP by carrying out knock down experiments as demonstrated in (Figure 7). But the persistence of transcriptional activity of rDNA in haploid spermatids although at lower level, raises an important question on the role of this rDNA transcriptional activity. It is quite plausible that this transcriptional activity may prevent any epigenetic modification at the rDNA locus of the germ cells during differentiation and maturation in the testis.

Another major interesting observation we have made in the present investigation is the genomic organization of the NRP gene. In contrast to the nucleolin gene which has 14 exons and 13 introns, the NRP gene has only 3 exons and 2 introns. There is a fusion of exons 5 through 14 of nucleolin gene generating a long exon 3 in the NRP gene. It is also interesting to note that the 3' UTR of both the nucleolin and NRP gene is identical. A detailed bioinformatics analysis of other mammalian genomes revealed that there are 2 additional NRP genes in addition to regular nucleolin gene in humans and 4 NRP genes in *Canis familiaris *(Figure 6B). The existence of these NRPs in other mammalian species as well, suggests that they might have evolved to attain new functions that needs to be explored further. The loss of N terminal acidic stretch the rat NRP might have a unique function in terms of its interacting partners compared to somatic nucleolin. The roles of these additional NRP genes present in these other mammalian species in the rDNA transcription needs to be investigated further to understand the functional significance of NRPs.

In addition to possible role(s) of the NRPs in ribosome biogenesis, it is quite likely that they may have completely different biological roles which need to be discovered. Thus our observations suggest that, other than supporting some functions of nucleolin, this variant may have a more specific role in meiotic prophase spermatocytes and haploid germ cells like *Modulo*, a *Drosophila *homologue of nucleolin [31]. They have shown that a testis specific variant of *Modulo *regulates the expression of meiotic arrest genes and is essential for transcription of spermatid differentiation genes. Surprisingly, we do find that the NRP gene is upregulated by several fold both in the meiotic spermatocytes and spermatid cells as compared to gametic diploid cells. Therefore it remains to be seen whether the mammalian *nucleolin *variant, *NRP *which is dramatically up regulated post meiosis, in the absence of nucleolin has a similar role as that of testis specific *Modulo *of *Drosophila *regulating expression of a set of genes during spermatogenesis. Further experiments are underway in this direction.

## Conclusion

In this report we identified a new variant of nucleolin (NRP) in testicular germ cells. We found that nucleolin is down regulated during spermatogenesis and this new variant has the potential to support rDNA transcription. We expect that these findings will be useful to better understand not only the existing variants of nucleolin in different cell types but also for exploring the functional significance of this new variant during spermatogenesis other than rDNA transcription.

## Methods

### Preparation of testicular cells, RNA isolation and Real time PCR

Diploids, meiotic spermatocytes and spermatid cells were isolated from the total testicular cells by the method of centrifugal elutriation as described previously by [44] using the elutriation rotor (JE 5.0) in a Beckman coulter centrifuge (Avanti J20-XP1). The collected cells were washed with 1× DPBS (Dulbecco's phosphate buffer saline). Aliquots of cellular fractions were fixed in 70% ice-cold ethanol for analysis by FACS. Cells for FACS analysis were stained using staining buffer containing (EtBr 25 μg/ml, 0.3%NP40 and 50 μg/ml of RNase A). After incubation at 37°C for 45 minutes, the purity of fraction was assessed using FACS (BD FACS calibur). Testicular cells from 10 day old rats were used as a source of gametic diploid cells.

RNA was isolated from diploid, meiotic spermatocytes and spermatid cells by the TRIzol reagent as per manufacturer's protocol. cDNA was synthesized from RNA of 10 day old rat testis, meiotic spermatocytes and spermatid cells using 2 μg of RNA (denatured and snap chilled), oligo (dT) 23 primer (Sigma), RT-buffer [50 mM Tris-HCl (pH 8.3), 75 mM KCl, 3 mM MgCl_2_, 10 mM dithiothreitol], dNTPs (1 mM each of dATP, dGTP, dTTP and dCTP) and 200 U of AMV Reverse transcriptase (NEB) according to the method as described in [45]. An aliquot of the RT-product was used for realtime PCR analysis of various genes using specific forward and reverse primers (given in additional file 1). Expression of Naca 1 gene was used as a normalizing control.

### DNA sequence analysis

PCR amplification of full-length *nucleolin *from total testicular cDNA using specific 5' and 3' end primers generated two amplicons. Bands were gel eluted using Qiagen gel elution kit, sequenced by ABI Prism 3100 sequencer and analyzed. Similarly gene sequence (3 kb including the 500 bp upstream of 5'UTR) of NRP was also amplified and sequenced.

### Evolutionary analysis using bioinformatic tools

The gene sequence and the mRNA sequence of NRP was analyzed for its conservation across Mouse and Rat using NCBI and Ensemble database. BLAST search was used for curating the sequences similar to NRP across the species. Clustal W was used for the alignment of sequences similar to NRP in different species for generating the dendrogram and studying the phylogenetic relationship. Proximal promoter region and the regulatory elements of both the *nucleolin *and *NRP *were analyzed using software Promoter scan  and TransFac search .

### Northern blot analysis

Northern blot was carried out using the MTN blot (Clontech) as per the manufacturer's protocol. Amplified full length PCR product of nucleolin as well as a specific portion (spanning the acidic stretch of the N-terminal region) of nucleolin was used for probe generation by Klenow labelling with α^32^P dATP along with random primers. Blots after washes were finally exposed to phosphor imager.

### Immunolocalisation and Immunoblotting of proteins

Total testicular cell smears were fixed with 4% paraformaldehyde (Sigma) for 15 minutes followed by permeabilization with 0.1% Triton X-100 (Sigma). Bovine serum albumin (1%) in PBS was used for blocking. Mouse monoclonal anti-nucleolin, rabbit polyclonal anti-UBF, goat polyclonal anti-fibrillarin (SantaCruz) were used for the detection of nucleolin, UBF and fibrillarin respectively. Colocalisation experiments were carried out by sequentially incubating the slides with each of the antibodies of nucleolin (rabbit polyclonal) or c-Myc (mouse monoclonal), BrUTP (mouse monoclonal) or UBF (rabbit polyclonal). Corresponding secondary antibodies conjugated with Alexa fluor dyes were used in each of the above experiments. Nuclei were stained with DAPI (Sigma). Images were acquired in a Ziess confocal laser scanning (LSM 510 META; Carl Zeiss).

For western blot analysis, nuclear or cell lysates were resolved in SDS-PAGE and electrophoretically transferred onto nitrocellulose membranes. Blocking was carried out using 5% skim milk powder in PBS followed by incubating with the appropriate primary antibody for 1 hour. The blot, after the washes was subsequently incubated with corresponding secondary antibody conjugated to HRP for 1 hour. The membrane was then washed and after the addition of substrate, chemiluminescence was captured by autoradiography.

### Nuclear run-on transcription and *In situ *run on transcription

*In vitro *nuclear run on transcription was carried out according to the method described [46] with minor modifications. Nuclei were isolated from the elutriated cells as described earlier. The elutriated cell pellet was treated with lysis buffer (10 mM Tris-HCl pH 7.4, 3 mM MgCl_2_, 10 mM NaCl, 0.1% NP-40) and incubated on ice for 15 minutes. The mixture was centrifuged at 4°C for 10 minutes at 1000 g. The supernatant was discarded and the nuclear pellet was resuspended in 120 μl glycerol buffer (50 mM Tirs-HCl pH 8.3, 5 mM MgCl2, 40% glycerol) and was mixed with equal volumes of 2× reaction buffer (10 mM Tris-HCl pH 7.0, 5 mM MgCl2, 300 mM KCl, 1 mM each of GTP, ATP, CTP and 80 μCi [μ32P]UTP, 1 mM DTT, 100 units of RNasin and 100 μg/ml of α-amanitin) the reaction was carried out for 30 minutes at 30°C. RNA was then isolated by using TRIzol reagent.

Dot blot for the run on transcription assay was prepared by blotting 800 ng of precipitated PCR amplicon products of 18S, 28S, ETS, ITS, Nucleolin and βActin were amplified using specific primer pairs (1–6) respectively (see additional file 1). The membrane was then dried and cross-linked under UV. The prehybridisation buffer (5× Denhardt's reagent, 6× SSC, 0.5% SDS, 100 μg/ml salmon sperm DNA, and 50% formamide) was added to the membrane and incubated at 42°C for 1–2 hours. The labelled transcribed RNA was hybridized for 48 hours at 42°C, followed by washing twice in 2× SSC, 0.5% SDS for 15 minutes, once in 1× SSC, 0.1% SDS for 15 minutes, and once in 0.5× SSC, 0.1% SDS for 15 minutes. The blot was then exposed to phosphor imager. The radioactivity incorporated in the blots was calculated densitometrically using Fugi image analysis software.

For *in situ *run-on transcription cells were resuspended in TBS (150 mM NaCl, 10 mM TrisCl (7.4), 5 mM MgCl2 containing 0.5% BSA), were washed twice with TBS and glycerol buffer (20 mM TrisCl (7.4), 5 mM MgCl2, 25% glycerol, 0.5 mM PMSF, 0.5 mM EGTA) and permeabilised in glycerol buffer containing 0.05% TritonX-100 for 5–10 minutes at room temperature. Permeabilized cells were washed once in TBS and run on transcription was performed in transcription buffer (100 mM KCl, 50 mM TrisCl (7.4), 5 mM MgCl2, 0.5 mM EGTA, 25% glycerol, 5 U/ml RNasin, 1 mM PMSF, 1 mM ATP, GTP, CTP, 0.5 mM BrUTP and 100 μg/ml of α-amanitin) for 30 minutes at 37°C. TCA and BSA was used to stop the reaction after 30 minutes and kept on ice for 30 minutes. Cells were then pelleted down and smears were made on microscope slides and fixed in 4% paraformaldehyde. Cells were again permeabilised with 0.1% TritonX-100 for 5–10 minutes. Smears were then blocked with 1% BSA for 45 minutes. Appropriate dilution of anti-BrdU antibody (Santa Cruz) was used as the primary antibody followed by incubation with secondary antibody conjugated with Alexa 568 for 1 hour at room temperature. Nuclei were stained with DAPI (1 μg/ml).

### Cell culture and transfections

GC1 Spg cells derived from B type spermatogonial cells, obtained from ATCC were cultured in DMEM (Sigma) supplemented with 10% FBS and appropriate antibiotics were used for all cellular assays. Cells were plated to 70% confluence at the time of transfection. Transfection was carried out using lipofectamine 2000 (Invitrogen) as per manufactures protocol with plasmid construct (NRP cloned in pCMV MYC vector). Cells were harvested at 48 or 60 hours post transfection and Immunoflourescence was carried out as described previously. Nucleolin smart pool siRNA was obtained from Dharmacon. GC1-Spg cells were cultured in 6 well plate and transfection was carried out as per the manufacturer's instructions. The nucleolin downregulated cells after transfection were then used for further experiments. Specific down regulation of nucleolin was carried out using antisense oligo's (oligo a (aacttcttcctcagagtcatcttc) and oligo b (cctcctcctcggccacactcttgg) spanning the exon 4 of nucleolin). Immunolocalisation and run on experiments were carried out after 60 hrs of transfection for the functional assays.

## Authors' contributions

TCK and GG participated in the design of the experiments and carrying out the experiment and analysis. MRSR conceived the study, and participated in its design and coordination and draft the manuscript. All authors read and approved the final manuscript.

## Supplementary Material

Additional file 1 **Primer Sequences**. Sequences of primer pairs used for different experiments are given. Click here for file
