# Supplementary material for: Identification of a novel nucleolin related protein (NRP) gene expressed during rat spermatogenesis
Source: BMC Mol Biol. 2009 Jul 1;10:64. doi: 10.1186/1471-2199-10-64 (PMC2711064; doi:10.1186/1471-2199-10-64)
Supplement: Additional file 1 — Primer Sequences. Sequences of primer pairs used for different experiments are given. [file 1471-2199-10-64-S1.doc]

| **Sl. No** | **Gene specific amplicon** | **Forward primer (5'-3')** | **Reverse primer(5'-3')** | **Accession**  **number of gene** | **Amplicon**  **Size (bp)** | **Experiments carried out with PCR amplicon** |
| --- | --- | --- | --- | --- | --- | --- |
| 1 | 18SrRNA | GAGGTTCGAAGACGATCAGA | TCGCTCCACCAACTAAGAAC | X01117 | 317 | Run on transcription |
| 2 | 28SrRNA | GGTACACCTGTCAAACGGTA | ACACCAAATGTCTGAACCTG | X00525 | 435 | Run on transcription |
| 3 | ETS | GTCTGAGAAGCCCGTGAGAG | GGGAAACCAGAAGACCAACA | X82564 | 317 | Run on transcription |
| 4 | ITS | CTGCTCTGGTCGAGGTTG | GTGAAGGAGAAGCGGAGA | X82564 | 404 | Run on transcription |
| 5 | Nucleolin (specific acidic stretch absent in NLP) | GCAAAAAGGCTACCACAACT | CATCTTCCTCATCCTCATCC | NM_ 012749 | 426 | Run on transcription and real time PCR |
| 6 | βactin | TTCTTTGCAGCTCCTTCGTTG | TGGATGGCTACGTACATGGCT | NM_007393.3 | 455 | Run on transcription |
| 7 | UBF1 | AGGCTGCAGAAGACCAAAAA | TTAGTTCCCCATTGGACAGC | M61726 | 168 | Real time PCR |
| 8 | L5 | TGGCCTGACAAACTATGCTG | GTAGACTGGATTCTCTCGGATAGC | NM_031099.1 | 490 | Real time PCR |
| 9 | S19 | ACGATGCCTGGAGTTACTGT | ATCCTGTCCAGATCTCTCTGTC | NM_001037346 | 401 | Real time PCR |
| 10 | rrp | TAAAAGCAAACCCACCATCC | CTCCTTGTCCCAGTCCTTCA | NM_026041 | 477 | Real time PCR |
| 11 | Ddx | TGTTCTCAGCCACACTGACC | AATGCCTCGAGCAGTAGCAT | NM_027156.2 | 425 | Real time PCR |
| 12 | Nucleolin mRNA full length | ATGGTGAAACTCGCAAAGGCCGGCAAAACCCACGG | TATTCAAACTTCGTCTTCTTTCCTTGTGGCTTGAAGTCTCC | NM_012749 | 2000 | Full length amplification and sequencing |
| 13 | NLP gene | GATTCTGGGGTCATTATTTTGTTCATGAAGGTT | AAGGCCAGCAGTGTAGCTCTATGGTAACGTG | *RGSC3, 4:15:3922898:3925784 | 3000 | Gene amplification and sequencing |
| 14 | Nucleolin gene | AGGAAGGTAACCAGATTAACT | AGTTACCCAGACTCTTTTACATACCTTTCT | NC_005108.2 | 1000 (of 8kb gene) | Gene amplification and sequencing |
| 15 | Fibrillarin | GGCGTAGACCAGATCCACAT | CAGCGTGGTCTCGTTCATAA | BC099198 | 480 | Real time PCR |

Additional File 1 : Primer sequences
